# Quantitative Determination of Acrolein in Cider by ^1^H NMR Spectrometry

**DOI:** 10.3390/foods9121820

**Published:** 2020-12-08

**Authors:** Enaitz de las Heras, Andoni Zuriarrain-Ocio, Juan Zuriarrain, Ane Bordagaray, María Teresa Dueñas, Iñaki Berregi

**Affiliations:** Faculty of Chemistry, University of the Basque Country EHU/UPV, 20018 Donostia-San Sebastián, Spain; enaitz_19@hotmail.com (E.d.l.H.); Zurimullen@hotmail.com (A.Z.-O.); zurijuan@hotmail.com (J.Z.); ane.bordagaray@ehu.eus (A.B.); mariateresa.duenas@ehu.eus (M.T.D.)

**Keywords:** acrolein, cider, nuclear magnetic resonance

## Abstract

Acrolein occasionally appears in cider, completely spoiling its quality due to its bitter taste. It is crucial to detect it in the early steps, before the taste is severely affected, to apply the appropriate treatment. A simple and rapid analytical method to determine this compound in cider is therefore desirable. In this work, a quantitative determination method of acrolein in cider is proposed using the proton nuclear magnetic resonance technique (^1^H NMR). Acrolein produces a doublet signal in the spectrum at 9.49 ppm, whose area is used to determine the concentration of this compound. 3-(trimethylsilyl)-2,2,3,3-d^4^-propionic acid sodium salt is added to the cider as a reference for 0.00 ppm and 1,3,5-benzenetricarboxylic acid as an internal standard for acrolein determination. The method is validated by gas chromatography (GC). There is a good correlation between the acrolein concentrations obtained by ^1^H NMR and by gas chromatography in different commercial ciders (Pearson coefficient 0.9994). The 95% confidence interval for the intercept is 0.15 ± 0.49 (includes 0) and for the slope is 0.98 ± 0.03 (includes 1). When applying the paired *t* test, no significant difference is observed. The proposed method is direct, and no prior derivatization is needed.

## 1. Introduction

Acrolein, 2-propenal or acrylaldehyde (CH_2_=CH–CHO), is a clear, colorless liquid with an intensively acrid odor. It is toxic to the lungs and irritating to mucous membranes. The compound is naturally emitted to the atmosphere as a product of fermentation and ripening processes. It is also released by forest fires as an incomplete combustion product. Anthropogenic sources such as pulp and paper mills, motor vehicles, waste incineration and coal-based electric power generation plants are also among the common sources of acrolein [1,2].

Acrolein is a compound that occasionally arises in cider, completely spoiling its quality due to its bitter taste. It is produced from glycerol by *Lactobacillus* bacteria through the intermediate product 3-hydroxy-propionaldehyde [3,4]. Although the presence of acrolein in apple juice derivatives was detected earlier [5], it was not until 2000 that it began to be studied in depth in these beverages [6,7].

Acrolein is a serious problem in cider of the Basque Country (northern Spain) as it appears every year in many cider factories. Basque cidermakers usually do not add sulfite to the fermenting musts until the end of alcoholic and malolactic fermentations. However, the formation of acrolein often occurs before this addition. At the present moment, when acrolein is detected in cider in concentrations lower than 5 mg/L, oenologists in the Basque Country simply add sulfite in a 20 mg/L concentration. If it is greater than 5 mg/L, the treatment used is more drastic: adding sulfite in a 120 mg/L concentration (sometimes even more, reaching to 150 mg/L), racking the cider (moving it to a clean tank to remove the sediments), adding the lees of a healthy fermenting must and stirring the mixture for 30 min. These treatments lower the acrolein concentration, so it is crucial to detect acrolein in the early steps before the taste is severely affected. For this reason, it is vital to have a simple and fast analytical method to determine acrolein in cider.

In beverages, acrolein is usually determined using gas chromatography [8,9,10,11,12], although high performance liquid chromatography (HPLC) methods [13,14,15] and some others [16,17] can also be found. The gas chromatographic methods have very low limits of detection (µg/L) because they include headspace solid-phase extraction or very sensitive detectors such as nitrogen–phosphorus. However, the method for determination of acrolein in Basque ciders should be rapid and simple, and the chromatographic methods do not fulfil these criteria. On the one hand, the headspace solid-phase extraction and desorption process takes no less than 20–25 min; on the other, most of these methods include a derivatization of acrolein, which usually takes 45 min or more.

The nuclear magnetic resonance (NMR) technique can detect a large number of components simultaneously in complex mixtures. In addition, sample preparation for NMR spectroscopy is simpler and less time consuming than for chromatography [18,19,20]. The purpose of this work is to provide a simple and fast procedure to determine acrolein in cider by proton nuclear magnetic resonance (^1^H NMR), in the same way that our research group has already achieved with other analytes, for example, caffeine, trigonelline, formic acid, and 5-(hydroxymethyl)furfural in soluble coffees [21].

## 2. Materials and Methods 

### 2.1. Chemicals and Reagents

The chemical reagents used were of analytical grade, and solutions were prepared with twice-distilled water (from here on out, “water”).

To begin, 1,3,5-Benzenetricarboxylic acid (≥95%) (BTC) was supplied by Merck (Darmstadt, Germany). Hydrochloric acid 37% (HCl), hydroquinone and sodium hydroxide (NaOH) were obtained from PanReac Química (Castellar del Vallès, Barcelona, Spain). Acetonitrile, acrolein (≥95.0%, ~0.2% hydroquinone as stabilizer), deuterium oxide (D_2_O), and 3-(trimethylsilyl)-2,2,3,3-tetradeuteropropionic acid sodium salt (TSP) were purchased from Sigma-Aldrich (St. Louis, MO, USA). 

### 2.2. Preparing the TSP-BTC-D_2_O Solution

First, 0.1053 g BTC (≥95%) was dissolved in 10–15 mL NaOH 1 M. The pH of the solution was adjusted to the 6–7 interval with HCl 0.5 M and diluted to 25 mL with water. BTC concentration: 4.001 g/L.

Next, 0.0100 g TSP was dissolved in 7 mL D_2_O, and 250 µL of the previous BTC solution was added. The solution was made up to 10 mL with water. Final concentrations: 1.00 g/L TSP, 0.1000 g/L BTC, and 70% (*v*/*v*) D_2_O.

### 2.3. Recording of ^1^H NMR Spectra: General Procedure

First, 600 µL of the calibration standard or the cider sample was placed in a 5 mm outer diameter NMR tube, and then 100 µL TSP-BTC-D_2_O solution was added. The final concentrations were TSP 0.143 g/L, BTC 0.0143 g/L and D_2_O 10% (*v*/*v*). D_2_O served as the field frequency lock, and all the spectra were referenced to the signal from TSP at δ = 0.00 ppm. BTC was added in exactly known concentration as an internal standard, which supplied a reference peak for the phenolic region of the spectrum (6–10 ppm).

The 500 MHz ^1^H NMR spectra were recorded at a temperature of 30 °C using a Bruker Avance 500 NEO spectrometer (Bruker, Billerica, MA, USA). A total of 128 scans of 78,124 data points were acquired with a spectral width of 7812 Hz (15.6 ppm), 4 dummy scans, acquisition time of 5.0 s, recycle delay of 5.0 s, flip angle of 90° and constant gain of 101, requiring 22.0 min per sample. Water suppression was performed by means of the WATERGATE pulse sequence [22]. 

Preliminary data processing was carried out with Bruker software, version 2.5. The Free Induction Decay signals were Fourier transformed (1.0 Hz line broadening), the spectra were phased, and the baseline was corrected. The resulting spectra were aligned by right or left shifting as necessary using the TSP signal as a reference. Data analysis was carried out with MestReNova 6.11-6384 software package [23].

### 2.4. Preparation of Stock Solution of Acrolein

By diluting the pure reagent, a 1000 mg/L stock solution of acrolein was prepared and stored in a refrigerator. Then, 0.2% hydroquinone was added to avoid oxidation and polymerization reactions. Pure acrolein is a toxic reagent with lachrymatory properties and must be handled always in a fume hood—wearing gloves, safety goggles and a protective mask. The bottle should be opened slowly so that the inner pressure decreases gently, and splashes are avoided. 

### 2.5. Calibration Graph

From the stock solution of acrolein, nine standards were prepared with concentrations in the range 5–50 mg/L, and their ^1^H NMR spectra were recorded according to the general procedure. To obtain the calibration graph, the ratio between the peak area of acrolein at 9.49 ppm and that of the internal standard BTC at 8.4–8.8 ppm was plotted against acrolein concentration. 

### 2.6. Determination of the Longitudinal Relaxation Time, T_1_

A volume of 5 μL pure acrolein (fume hood) and D_2_O up to about 700 µL were placed in a 5 mm outer diameter NMR tube (~114 mM acrolein). To measure the *T*_1_ of the aldehydic proton of acrolein, the longitudinal relaxation delay of the selected proton was determined by means of the inversion recovery pulse sequence method [24]. *T*_1_ cal Bruker program was used for this purpose. This program fits the data to the exponential equation *I = I*_0_ *+ P exp* (*−τ/T*_1_)*,* where *I* is the intensity of the proton resonance at inversion delay *τ*, *I*_0_ the intensity at the equilibrium state and *P* a constant. Inversion delays used were 2.00, 4.00, 8.00, 16.00, 30.00, 60.00, 90.00, 120.00 and 150.00 s. The standard deviation of *T*_1_ was obtained from the curve fit.

### 2.7. Preparing the Cider Samples

Eight commercial ciders of the Basque Country were used in this study. Each of them was homogenized by manual shaking, and a 50 mL fraction was degasified by using a stirrer and a vacuum pump. A small part of this solution (<2 mL) was immediately used for ^1^H NMR spectra recording and determination of acrolein with this technique. The rest of the solution was centrifuged at 8200 rpm (9000× *g*) for 20 min in a Thermo Scientific^TM^ Sorvall ST8 centrifuge (Thermo Fisher Scientific, Waltham, MA, USA). A fraction of this centrifuged sample was filtered through a 0.45 μm filter and distributed in two vials. The vials were stored in the freezer at −20 °C until the determination of acrolein by gas chromatography was performed.

### 2.8. Analysis of Samples by Gas Chromatography

Acrolein was determined using gas chromatography with an Agilent HP 6890N gas chromatograph (Agilent Technologies, Santa Clara, CA, USA), equipped with a flame ionization detector and fitted by a Restek-Stabilwax^®^ capillary column (60 m, 0.53 mm ID, 1 µm df) with polyethylene glycol internal coating (Restek Corporation, Bellefonte, PA, USA). Helium at 7 mL/min was used as the carrier gas.

The samples were thawed, 1 mL of each one was mixed with 100 μL of 800 mg/L acetonitrile, as an internal standard, and 3 µL of this mixture was injected in the split mode (ratio 15:1). The injector temperature was fixed at 200 °C, the pressure at 0.597 bar and the total flow rate at 115 mL/min. The detector temperature was fixed at 250 °C. Hydrogen (40 mL/min), air (450 mL/min) and nitrogen (20 mL/min) were used as detector gases. The oven temperature gradient was as follows: 1 min at 40 °C, an increase to 65 °C at 5 °C/min, 1 min at 65 °C, another increase to 125 °C at 15 °C/min and finally a 4 min post-run stage at 200 °C to clean the column. The temperature was then allowed to return to the initial value of 40 °C and 3 min more were given to homogenize the oven temperature. The total time for each chromatogram was 24 min (11 min measuring). Acrolein was quantified from the peak area. This method was used by our research team to determine in ciders not only acrolein but also acetaldehyde, ethyl acetate, methanol and ethanol. 

A calibration graph was obtained previously by using eight standards with acrolein concentrations in the range 5–50 mg/L, prepared from the stock solution. The stock solution and the standards were again prepared to be different from those used in ^1^H NMR calibration graph. Then, 1 mL of each standard was mixed with 100 μL of 800 mg/L acetonitrile, and 3 µL of this mixture was injected in the same way as the samples. 

## 3. Results

### 3.1. H NMR Spectra of Ciders

**Figure 1:**
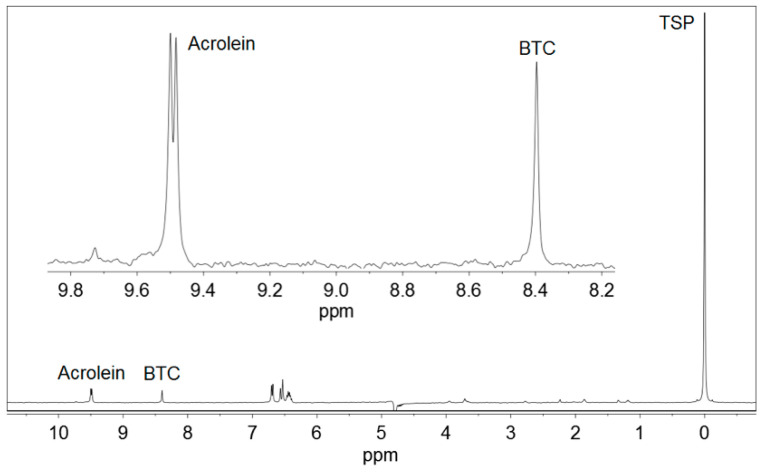
^1^H NMR spectrum of a standard for calibration containing 35.1 mg/L acrolein, 1,3,5-benzenetricarboxylic acid (BTC) and 3-(trimethylsilyl)-2,2,3,3-tetradeuteropropionic acid sodium salt (TSP). An expansion of the working region is shown.

**Figure 2:**
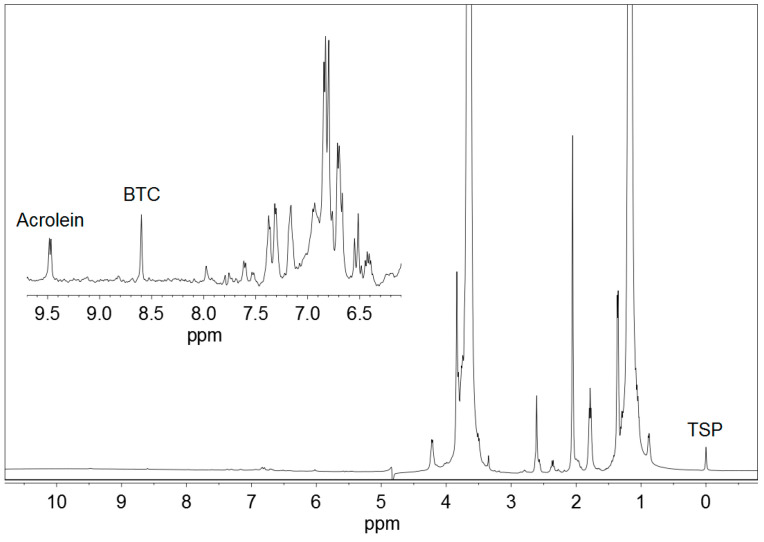
^1^H NMR spectrum of a commercial Basque cider containing 21.8 mg/L acrolein, 1,3,5-benzenetricarboxylic acid (BTC) and 3-(trimethylsilyl)-2,2,3,3-tetradeuteropropionic acid sodium salt (TSP). An expansion of the working region is shown.

Figure 1 displays the ^1^H NMR spectrum of a standard solution of acrolein, and Figure 2 represents a commercial Basque cider containing acrolein. BTC and TSP were added to both the samples. Other ciders produced qualitatively similar spectra. 

Acrolein (CH_2_=CH–CHO) gave a doublet signal at 9.49 ppm, corresponding to the proton from the aldehyde group –CHO, which was used for quantitative determinations. This signal moved very slightly with pH. It appeared at 9.48 ppm at pH < 1.0, moved to 9.49 ppm at pH 1.0–2.0, and stayed at 9.49 ppm at pH 2.0–5.0. As the pH of Basque ciders was usually between 3.5 and 4.0, no higher values of pH were studied. 

Acrolein gave also a double doublet signal at 6.55 ppm, produced by one of the protons of the CH_2_= group, and a mixed signal at 6.35 ppm, a combination of the double doublet produced by the other proton of the CH_2_= group and the multiplet produced by the proton of the =CH– group. All these signals were not used because they overlapped with others present in ^1^H NMR spectra of ciders, as can be depicted in Figure 2.

**Figure 3:**
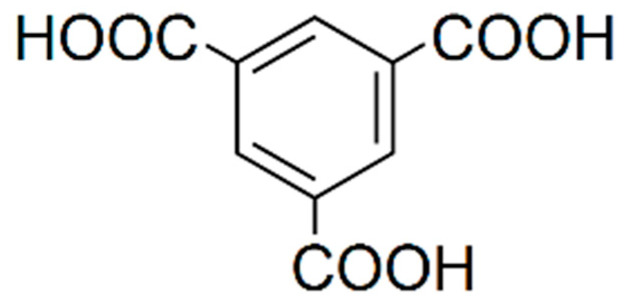
1,3,5-benzenetricarboxylic acid (BTC).

BTC (Figure 3) presented a singlet signal at 8.40–8.81 ppm, depending on the pH, corresponding to the three equivalent protons of the benzene ring. This signal was located at 8.81 ppm at pH ≤ 1.5, it moved to 8.40 in the pH interval of 1.5–4.1 and remained constant at 8.40 ppm in the pH range of 4.1–5.0 [25]. BTC was selected as an internal standard for the low field region of the spectra because it is not present in ciders. In addition, it is soluble in water and produces a strong and clear singlet signal, which never overlaps with any other signal of the ciders. The use of internal standards in quantitative ^1^H NMR is a common procedure used in many determination methods [26,27,28,29,30].

TSP ((CH_3_)_3_–Si–CD_2_–CD_2_–COONa) was soluble in water and had a simple ^1^H NMR spectrum with a single nine proton singlet, corresponding the three methyl groups –CH_3_. This signal is a common reference for 0.0 ppm chemical shift in the ^1^H NMR spectra performed in aqueous solutions.

The only chemical shift influenced significantly by pH was that of BTC, but this is not relevant as the signal did not overlap with any other signals. As a consequence, the pH did not require to be adjusted. 

### 3.2. Optimisation of ^1^H NMR Acquisition Conditions

The acrolein content in ciders is usually low, so the intensity of its signal must be increased to get a good sensitivity in the analytical determination. In order to obtain the highest signal intensity, the time between the successive acquisitions of the spectra should be long enough to guarantee that the protons involved are completely relaxed. This means that the sum of acquisition time and recycle delay time should be, at least, equal to the longitudinal relaxation time *T*_1_ of the nuclei involved in the signals. The *T*_1_ value for the proton of the –CHO group of acrolein (working proton), obtained from the inversion recovery pulse sequence method, was 15.348 ± 0.006 s. The *T*_1_ value for the three protons of BTC, obtained in a previous work, was 10.142 ± 0.007 s [25]. According to this, the critical factor is the relaxation time of the working proton of acrolein. On the other hand, another way to enhance the signal intensity is to increase the number of scans.

**Figure 4:**
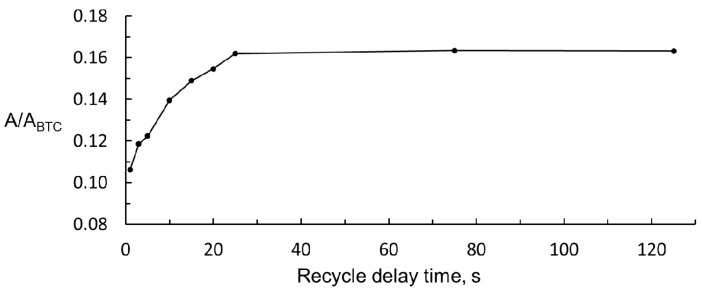
Change in acrolein relative signal with recycle delay time.

The acquisition time was fixed at 5.0 s to ensure good digital resolution to the spectra obtained; the number of scans fixed was 128 to ensure a good signal to noise relationship. The latter is particularly important in the low field region of the spectra of ciders because wake signals are obtained in this region, and the working signal of acrolein is not an exception. To find the best value of recycle delay time, many ^1^H NMR spectra of 50 mg/L solution of acrolein were performed, according to the general procedure but modifying the recycle delay time from 1 to 125 s. The ratio between peak areas of acrolein (A) and BTC (A_BTC_) was calculated for each spectrum. The results are shown in Figure 4. It can be seen that the acrolein relative signal reaches a maximum at a delay time of 25 s. If the total scan time was fixed at 30.0 s (acquisition time, 5.0 s, and delay time 25.0 s) and 128 scans plus four dummy scans were performed, more than one hour (66 min) would be needed to register each ^1^H NMR spectrum, which was considered too long. The determination method proposed should not be much longer than the 20 min needed for the chromatographic determination. For this reason, a 5 s delay time was chosen. With this value, the intensity of the relative signal of acrolein was still 75% of the maximum, and the ^1^H NMR spectrum was recorded in 22.0 min. 

### 3.3. Calibration Equation and Limit of Detection

To achieve the calibration graph for acrolein, the ratio between peak areas of acrolein (A) and the internal standard BTC (A_BTC_) was plotted against acrolein concentration (C, mg/L). The data were processed with Microsoft Excel 2016, and the following equation was obtained:

A/A_BTC_ = (5.72 ± 0.07) × 10^−2^ C − (2 ± 2) × 10^−2^

*N* = 9      *r* = 0.9995     S*_y_*_/*x*_ = 2.97 × 10^−2^

A high correlation coefficient was obtained, which indicates a good linearity response in the concentration range studied. This was confirmed using the *t* test [31], which gave a high *t* value (80.0) with a significance lower than 0.01. The limit of detection (LOD), obtained from “3 S*_y_*_/*x*_+ intercept”, was 1.6 mg/L.

### 3.4. Precision

The precision of the method was calculated with an intra-day repeatability test. Using the general procedure, ^1^H NMR spectrum of a 10.0 mg/L standard of acrolein was repeated 10 times on the same day and then, the concentration of acrolein was determined with the calibration equation. A coefficient of variation of 4.2% was achieved from these measurements. Due to the volatility of the compound, no measurements can be made on different days, so the inter-day repeatability was not evaluated. 

### 3.5. Application to Commercial Ciders

**Figure 5:**
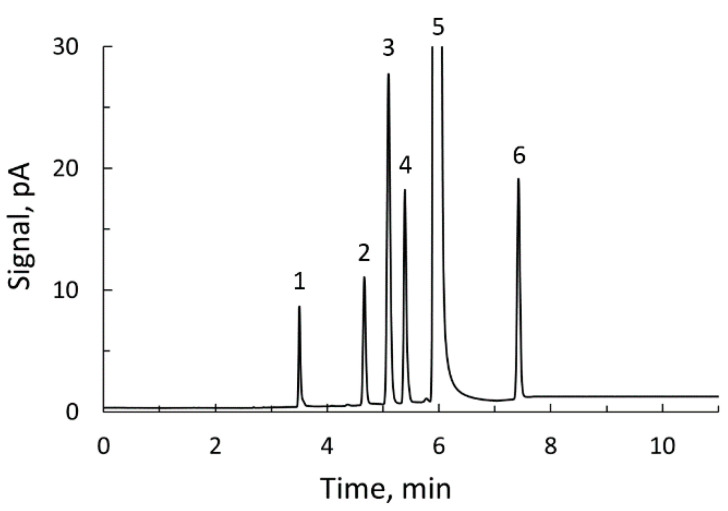
Gas chromatogram of a standard for determination of volatile compounds in ciders: 1, acetaldehyde; 2, acrolein; 3, ethyl acetate; 4, methanol; 5, ethanol; 6, acetonitrile (internal standard).

The ^1^H NMR procedure described was validated by using it in the determination of acrolein in eight cider samples containing this compound in different concentrations. For comparison purposes, acrolein was determined in the same eight commercial ciders by means of the gas chromatography method described before. Figure 5 shows the chromatogram obtained with a standard solution prepared for the determination of volatile compounds in ciders. In the chromatograms obtained with the cider samples, one or more of these peaks could be missing, except that of ethanol which always appears. With the cider samples selected for this work, the peak of acrolein was always present. The acrolein contents obtained with the two techniques are given in Table 1.

The results obtained by ^1^H NMR were plotted against those obtained by GC in the same samples. The data were fitted by the least-squares method, and a Pearson correlation coefficient of 0.9994 was obtained. The 95% confidence interval for the intercept was 0.15 ± 0.49, which includes the zero, and that for the slope was 0.98 ± 0.03, which includes the 1.0. The pair *t*-test was also used to compare both techniques giving a *t*-value of 0.78, while the critical *t*-value for a 95% confidence level was 2.36. These results indicate that no significant difference was found between the two methods, so the ^1^H NMR method described can be considered valid for the determination of acrolein in cider samples [31]. 

The method proposed can be useful to all cider producers who have an NMR service nearby, and this is the situation of most of the cidermakers of the Basque Country. The measurement time of 22.0 min was similar to that of the chromatographic method used, and shorter than those quoted in the introduction. The main drawback of the method is its high LOD of 1.6 mg/L, as many chromatographic methods offer LOD values in the order of µg/L. However, all these methods include preconcentration procedures such as headspace solid-phase extraction and long time periods for derivatization. The method required by cidermakers of the Basque Country should be direct, simple and rapid. Moreover, the tolerable oral acrolein concentration is 1.5 mg/L [1], almost the same as the LOD obtained here. Furthermore, there is no need for so low LOD values in Basque ciders. In fact, the Fraisoro Agro-environmental Laboratory (Zizurkil, Gipuzkoa, Basque Country), which is a reference in the analysis of Basque ciders, does not consider concentrations under 2 mg/L. Therefore, the ^1^H NMR method described here is sufficient for the proposed objective and faster than the sensitive chromatographic methods. In addition, it allows the simultaneous determination of many other important compounds present in ciders, whose methods by ^1^H NMR have been previously reported by our laboratory group: ethanol [32], malic acid [33], lactic acid and acetic acid [34], chlorogenic acid [35] and (−)-epicatechin [36]. 

## Figures and Tables

**Table 1:** Acrolein concentration in eight commercial ciders. Determined by ^1^H NMR and GC methods.

Cider	* Concentration, mg/L	
	^1^H NMR	GC
1	9.8 ± 0.2	10.3 ± 0.3
2	6.0 ± 0.1	5.7 ± 0.2
3	2.6 ± 0.1	2.5 ± 0.1
4	6.8 ± 0.2	6.7 ± 0.2
5	2.3 ± 0.1	2.4 ± 0.1
6	12.3 ± 0.3	12.8 ± 0.4
7	31.8 ± 0.7	32.5 ± 0.9
8	15.6 ± 0.4	15.2 ± 0.4

* Average of three measurements ± standard deviation.
